# Synthesis and Characterization of Nanoparticles in Transforming Biodiesel into a Sustainable Fuel

**DOI:** 10.3390/molecules30061352

**Published:** 2025-03-18

**Authors:** Ramozon Khujamberdiev, Haeng Muk Cho

**Affiliations:** Department of Mechanical Engineering, Kongju National University, Cheonan 31080, Republic of Korea; khujamberdievramozon@gmail.com

**Keywords:** nanoparticles, biofuels, combustion efficiency, emission reduction, hybrid nanomaterials, sustainable fuel

## Abstract

Biodiesel is a promising alternative to fossil fuels, offering environmental benefits but facing challenges such as low energy density, poor oxidative stability, and high emissions. Nanotechnology has emerged as a solution, with nanoparticles improving biodiesel properties. This review examines the synthesis, characterization, and application of metal-based, carbon-based, and hybrid nanomaterials in biodiesel. Notable enhancements include an 18% increase in brake thermal efficiency with aluminum oxide and a 20% reduction in NOx emissions with cerium oxide. Hybrid nanoparticles, like graphene oxide with carbon nanotubes, have achieved a 25% decrease in hydrocarbon emissions. Despite these advancements, concerns regarding nanoparticle toxicity, environmental impact, and stability remain. Future research should focus on eco-friendly synthesis, integration with second-generation biodiesel, and multifunctional hybrid nanomaterials. This review highlights the potential of nanotechnology in enhancing biodiesel performance, paving the way for cleaner and more efficient energy solutions.

## 1. Introduction

### 1.1. Background and Significance

Biodiesel has gained recognition as a sustainable alternative to conventional fossil fuels, meeting the increasing global energy demand while reducing the environmental impact. Sourced from renewable biological materials such as vegetable oils and animal fats, it offers advantages like biodegradability, non-toxicity, and lower greenhouse gas emissions. However, its widespread adoption faces challenges related to engine performance, fuel stability, and emissions. Compared to petroleum diesel, biodiesel’s higher viscosity, lower energy content, and oxidative instability can lead to increased engine wear, inefficient combustion, and elevated nitrogen oxide (NOx) emissions [1].

To address these limitations, advancements in nanotechnology have provided innovative solutions, enhancing the properties of biodiesel through the incorporation of nanoparticles as additives or catalysts. These nanoparticles, including materials like titanium dioxide (TiO_2_) and cerium oxide (CeO_2_), play a significant role in improving fuel performance by optimizing combustion characteristics, reducing emissions, and increasing thermal efficiency [2]. Moreover, their catalytic properties facilitate more efficient biodiesel production processes, reducing energy costs and enhancing yield [3].

However, biodiesel is not without its challenges. Among its key drawbacks are increased NOx emissions, lower oxidative stability, and inconsistencies in fuel properties, such as viscosity, density, and calorific value, which can negatively impact engine performance and durability [4]. Additionally, the chemical composition of biodiesel, which varies depending on the feedstock, makes it prone to degradation during storage and operation under extreme conditions, further limiting its long-term viability as a diesel substitute [5]. Addressing these issues is critical for enhancing biodiesel’s competitiveness in the global fuel market.

Nanotechnology has emerged as a transformative approach to overcoming these limitations. Nanoparticles, owing to their exceptional surface-area-to-volume ratio, high reactivity, and unique catalytic properties, have demonstrated the ability to enhance the combustion efficiency and overall performance of biodiesel. For example, metal oxide nanoparticles such as titanium dioxide (TiO_2_) and cerium oxide (CeO_2_) have been shown to improve fuel atomization, increase energy release during combustion, and reduce NOx and CO emissions [6,7]. Additionally, nanoparticles enhance the oxidative stability of biodiesel, mitigating issues related to fuel degradation and ensuring better engine performance over time [8].

As the world transitions toward renewable energy solutions, integrating nanotechnology into biodiesel synthesis and characterization not only addresses technical challenges but also paves the way for more sustainable and efficient energy systems. This review delves into the latest developments in nanoparticle applications in biodiesel production, focusing on their synthesis, characterization, and impact on fuel properties and engine performance.

Recent studies highlight the potential of nanoparticle additives not only in addressing the technical challenges of biodiesel but also in opening new avenues for optimizing its properties. However, while significant progress has been made in demonstrating their effectiveness, understanding the chemical synthesis and characterization of nanoparticles specifically tailored for biodiesel applications remains underexplored. This review aims to address this gap by focusing on the synthesis techniques, physicochemical properties, and chemical interactions of nanoparticles used in biodiesel. By providing a comprehensive overview, this review seeks to contribute to the ongoing development of nanotechnology-driven solutions for cleaner and more sustainable energy.

### 1.2. Scope of the Review

This review focuses on the synthesis, characterization, and application of nanoparticles within the context of biodiesel production and utilization. The integration of nanoparticles into biodiesel systems represents a groundbreaking approach to overcoming the technical and environmental challenges associated with biodiesel as an alternative fuel. By enhancing critical properties such as combustion efficiency, fuel stability, and emission reduction, nanoparticles have emerged as vital enablers of biodiesel performance improvement [9].

The scope of this review extends across three major domains:

1. The synthesis of nanoparticles for biodiesel applications

The review explores state-of-the-art methods employed in nanoparticle synthesis, including sol–gel techniques, hydrothermal methods, and green synthesis approaches. These methodologies are critical for tailoring nanoparticle properties to achieving specific biodiesel enhancements.

2. The characterization of nanoparticles

Detailed characterization techniques, such as scanning electron microscopy (SEM), transmission electron microscopy (TEM), and X-ray diffraction (XRD), are discussed for evaluating the structural, thermal, and catalytic properties of nanoparticles. These properties directly influence the efficiency and sustainability of biodiesel production and application.

3. The application of nanoparticles in biodiesel

The practical use of nanoparticles as fuel additives, catalysts, and performance enhancers in biodiesel systems is critically reviewed. Emphasis is placed on their role in improving fuel combustion, reducing greenhouse gas emissions, and enhancing overall energy efficiency.

The review highlights the relevance of these advancements to the global pursuit of energy sustainability. As the demand for renewable energy sources grows, the integration of nanotechnology into biodiesel systems represents a promising pathway to achieving a more sustainable and energy-efficient future. This review aims to provide a comprehensive overview of the scientific progress and practical applications of nanoparticles in biodiesel, offering insights into their potential in revolutionizing the biofuel industry.

### 1.3. Materials and Methods

This review was conducted by systematically analyzing the existing literature on nanoparticles and biofuels. Relevant research articles were sourced from databases such as Google Scholar, Scopus, and Web of Science, using keywords including nanoparticles, hybrid nanomaterials, biofuels, and emissions. Studies were selected based on their relevance, recent advancements, and contributions to the field.

To assist in organizing and summarizing the reviewed literature, ChatGPT-3.5 (OpenAI, free version) was used in a limited capacity. Specifically, it was employed to generate concise summaries of publicly available research papers to facilitate the literature review process. Additionally, it was used to refine grammar, sentence structure, spelling, punctuation, and formatting. The AI tool was not used for generating original research content, analyzing data, or drawing conclusions. All AI-assisted outputs were carefully reviewed and verified by the authors to ensure accuracy and compliance with ethical standards.

## 2. Nanoparticles in Biodiesel: Overview

### 2.1. Types of Nanoparticles Used in Biodiesel

Nanoparticles have garnered significant attention for their ability to enhance biodiesel’s performance and mitigate its limitations. The incorporation of nanoparticles into biodiesel has shown promise in improving combustion efficiency, reducing emissions, and enhancing fuel stability. This section provides an overview of the types of nanoparticles commonly utilized in biodiesel applications, including metal-based, carbon-based, and hybrid/composite nanoparticles.

#### 2.1.1. Metal-Based Nanoparticles

Metal-based nanoparticles, including titanium dioxide (TiO_2_), aluminum oxide (Al_2_O_3_), zinc oxide (ZnO), and cerium oxide (CeO_2_), have emerged as pivotal additives in biodiesel for improving combustion efficiency, fuel stability, and emission characteristics. Their catalytic properties and high surface-area-to-volume ratios significantly enhance the fuel’s thermophysical behavior, making them effective in addressing biodiesel’s inherent limitations.

Titanium dioxide (TiO_2_): TiO_2_ nanoparticles have been widely studied for their role in enhancing combustion and emission performance. They act as oxygen donors, promoting complete combustion, which leads to reduced carbon monoxide (CO) and unburned hydrocarbon (HC) emissions. Additionally, they improve the oxidation stability of biodiesel, which is critical for its storage and long-term usability. However, their high production cost and potential nanoparticle agglomeration can negatively affect fuel homogeneity and injector performance if not properly dispersed [10].

Aluminum oxide (Al_2_O_3_): Al_2_O_3_ nanoparticles are particularly effective in enhancing brake thermal efficiency (BTE) and reducing emissions. Studies have demonstrated significant improvements in combustion efficiency and reductions in harmful exhaust emissions such as CO and HC when biodiesel is blended with Al_2_O_3_ nanoparticles. Despite these benefits, high concentrations of Al_2_O_3_ may lead to increased fuel viscosity and deposition issues within the engine, affecting long-term performance [11,12].

Zinc oxide (ZnO): ZnO nanoparticles enhance biodiesel’s thermal stability, making it suitable for high-temperature operations. Their inclusion has been shown to increase fuel efficiency and reduce emissions of particulate matter and NOx. Additionally, ZnO nanoparticles provide a synergistic effect when combined with other additives, further improving engine performance. But ZnO’s limited solubility in biodiesel poses challenges, often requiring surfactants or dispersion techniques to ensure uniform mixing [13,14].

Cerium oxide (CeO_2_): CeO_2_ nanoparticles are particularly noteworthy for their oxygen storage and release capabilities, which facilitate complete combustion and lower particulate matter emissions. These nanoparticles also contribute to reducing soot formation and improving the oxidative characteristics of biodiesel. However, concerns about long-term nanoparticle accumulation in engine components raise questions about potential wear and deposit formation, necessitating further investigation into their long-term effects on engine health [15,16].

Studies combining multiple metal-based nanoparticles, such as ZnO and Al_2_O_3_, show synergistic effects in enhancing fuel properties and reducing emissions. Hybrid nanofuels containing such blends have achieved significant improvements in combustion characteristics, such as a higher BTE and reduced NOx emissions [17]. By integrating metal-based nanoparticles, biodiesel blends can achieve superior combustion efficiency, lower emissions, and enhanced thermal stability.

#### 2.1.2. Carbon-Based Nanoparticles

Carbon-based nanoparticles, such as graphene, graphene oxide (GO), and carbon nanotubes (CNTs), are widely recognized for their superior thermal conductivity, extensive specific surface area, and excellent mechanical strength. These characteristics make them effective in improving combustion efficiency and emission profiles in biodiesel applications. These nanoparticles can enhance fuel atomization and enable faster combustion cycles, ultimately contributing to better engine performance and lower emissions.

Graphene and Graphene Oxide (GO): Graphene and GO nanoparticles exhibit high thermal conductivity and excellent stability, enabling significant improvements in biodiesel combustion. Studies have demonstrated that adding GO to biodiesel blends enhances brake thermal efficiency (BTE) and reduces NOx emissions by accelerating combustion cycles [18]. Additionally, surfactant-stabilized GO nanoparticles have been shown to improve the thermal and emission properties of biodiesel blends, highlighting their potential as a viable additive. But the high cost of graphene-based nanomaterials, along with their tendency to agglomerate in biodiesel, presents challenges for large-scale implementation. Without proper dispersion techniques, such as ultrasonication or surfactant modification, their effectiveness may be reduced [19].

Carbon Nanotubes (CNTs): CNTs are another promising additive in biodiesel blends. Their high aspect ratio and thermal conductivity enhance fuel properties and improve engine performance. CNTs have demonstrated the ability to reduce CO and hydrocarbon (HC) emissions while enhancing ignition and combustion characteristics. For instance, the integration of multi-walled carbon nanotubes (MWCNTs) into biodiesel has shown significant reductions in emissions and improvements in brake-specific fuel consumption. Despite these advantages, CNTs’ poor solubility in biodiesel and high production costs pose significant challenges. Without effective functionalization or dispersion aids, CNTs tend to settle over time, leading to inconsistent fuel properties and potential clogging in fuel injection systems [20,21].

The combination of graphene and CNTs has shown a synergistic effect in further enhancing biodiesel performance. These hybrid formulations optimize thermal properties, emission characteristics, and overall efficiency of biodiesel engines [22]. Through the incorporation of these carbon-based nanoparticles, biodiesel blends achieve better combustion efficiency, reduced emissions, and enhanced fuel stability, positioning these nanomaterials as integral components of sustainable fuel innovation.

#### 2.1.3. Hybrid or Composite Nanoparticles

Hybrid nanoparticles integrate properties from different materials to achieve enhanced performance in biodiesel applications. A composite of TiO_2_ and Al_2_O_3_ has been shown to improve both thermal stability and combustion efficiency, making it a versatile choice for biodiesel additives [21]. Another example includes hybrid GO and carbon nanotubes (CNTs), which form stable biodiesel blends with significantly reduced emissions, leveraging the unique thermal conductivity and mechanical strength of these materials [22]. These composites require precise synthesis techniques to prevent agglomeration, which can limit their effectiveness and stability in fuel systems.

Recent advancements have introduced metal–organic frameworks (MOFs) hybridized with nanoparticles like silver to produce catalysts such as Ag@MOF-801. This hybrid not only boosts catalytic efficiency during transesterification but also lowers the energy requirements for biodiesel production [23]. Similarly, composites of ZnO, Al_2_O_3_, and TiO_2_ demonstrate enhanced thermal and oxidative stability, key for high-performance biodiesel fuels. However, their dispersion challenges in biodiesel necessitate functionalization or surfactant modifications to ensure uniform mixing [24].

The introduction of hybrid nanofluids, such as Al_2_O_3_–SiO_2_ composites, in diesel–biodiesel blends has been observed to optimize engine performance while reducing harmful emissions. Studies reveal improved brake thermal efficiency and lower brake-specific fuel consumption when using these hybrids as additives [25]. Nonetheless, the long-term effects of hybrid nanoparticle accumulation in engine components and their cost-effectiveness at industrial scale remain areas of concern.

**Figure 1:**
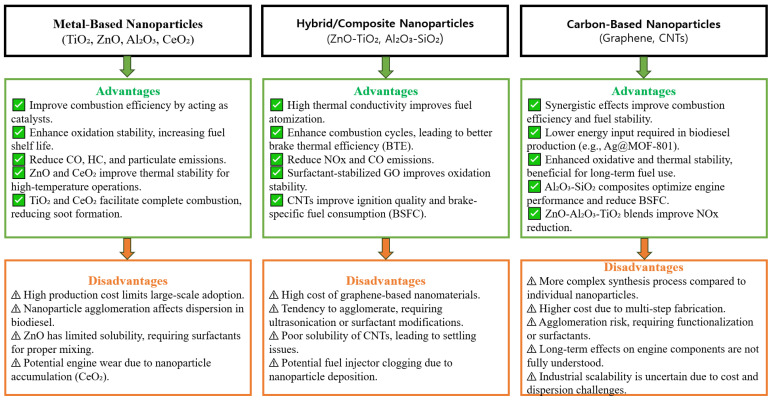
Advantages and disadvantages of nanoparticle categories in biodiesel.

Figure 1 shows a structured table outlining the advantages and disadvantages of metal-based, carbon-based, and hybrid/composite nanoparticles.

### 2.2. Advantages of Using Nanoparticles in Biodiesel

#### 2.2.1. Improved Combustion Efficiency

Nanoparticles play a critical role in enhancing the combustion efficiency of biodiesel by increasing the surface-area-to-volume ratio, which facilitates better air–fuel mixing and promotes a more complete combustion. Graphene oxide (GO) nanoparticles, when added to mahua biodiesel blends, have been shown to increase brake thermal efficiency (BTE) by approximately 5% while reducing brake-specific fuel consumption (BSFC) by 6% due to their ability to enhance fuel atomization and oxidation kinetics [19]. Similarly, TiO_2_ nanoparticles improve thermal efficiency and ignition delay, which collectively enhance the engine’s combustion profile by promoting faster and more efficient fuel oxidation [26].

The catalytic nature of metal oxide nanoparticles, such as aluminum oxide (Al_2_O_3_) and cerium oxide (CeO_2_), enables them to act as oxygen carriers, accelerating hydrocarbon oxidation and promoting a more uniform heat distribution within the combustion chamber. This process enhances ignition characteristics and flame propagation, leading to a higher brake thermal efficiency (BTE) and reduced brake-specific fuel consumption (BSFC) [27]. Additionally, nanoparticles reduce wall-wetting effects by improving the evaporation characteristics of biodiesel, ensuring a finer spray formation and better air–fuel mixing [28].

#### 2.2.2. Emission Reductions

The integration of nanoparticles into biodiesel blends significantly reduces harmful emissions, including carbon monoxide (CO), unburned hydrocarbons (UHC), NOx, and particulate matter (PM). ZnO nanoparticles, for instance, have demonstrated reductions in CO emissions by up to 8% and NOx emissions by approximately 11% when blended with waste frying oil biodiesel, due to their role in enhancing oxidation and controlling flame temperature fluctuations [29]. Similarly, cerium oxide nanoparticles, known for their oxygen storage and release capabilities, facilitate NOx reduction by acting as redox catalysts, converting NOx into nitrogen (N_2_) at lower temperatures, thereby mitigating thermal NOx formation [30].

Hybrid nanoparticles, such as graphene oxide combined with carbon nanotubes, create a synergistic effect, enabling substantial reductions in CO and HC emissions while maintaining a high combustion efficiency by improving turbulence and enhancing heat transfer rates [20]. Carbon-based nanoparticles, such as graphene oxide and carbon nanotubes, further improve thermal conductivity, ensuring a higher oxidation rate of CO to CO_2_, thereby reducing carbon monoxide emissions [31]. Moreover, cerium-coated ZnO nanoparticles in soybean biodiesel blends have been shown to increase BTE by 20.66% while significantly reducing CO and smoke opacity, demonstrating the effectiveness of nanoparticle-infused biodiesel in lowering emissions [32].

#### 2.2.3. Enhanced Thermal and Oxidative Stability

Nanoparticles also improve the thermal and oxidative stability of biodiesel, addressing one of its primary limitations: susceptibility to oxidation during storage. The addition of silica-coated nanoparticles, such as SiO_2_, enhances both thermal and oxidative stability, ensuring longer storage durations and optimizing engine performance during sustained use [33]. Similarly, palladium nanoparticles incorporated into biodiesel formulations increase oxidative stability, extending fuel shelf life and improving storage properties [34].

Additionally, ZnO and Al_2_O_3_ nanoparticles enhance thermal stability, allowing biodiesel to perform effectively under high-temperature conditions [35]. These nanoparticles prevent fuel degradation by inhibiting peroxide formation, which is responsible for oxidative instability. By mitigating oxidation-related degradation, nanoparticle-enhanced biodiesel maintains consistent fuel properties, ensuring reliable engine operation and reducing maintenance requirements [36].

## 3. Synthesis of Nanoparticles for Biodiesel Applications

### 3.1. Chemical Synthesis Methods

#### 3.1.1. Sol–Gel Method

The sol–gel process is a widely used chemical synthesis method for nanoparticles due to its ability to produce materials with a high purity and homogeneity. This method involves the transition of a solution system from a liquid “sol” into a solid “gel” phase. For example, TiO_2_ nanoparticles synthesized via sol–gel have shown excellent catalytic activity in biodiesel production, enhancing both the efficiency and stability of the biodiesel blends [37]. Furthermore, the sol–gel method has been employed for the synthesis of alumina nanoparticles, which significantly reduce emissions and improve biodiesel’s oxidative stability [38].

#### 3.1.2. Hydrothermal Method

The hydrothermal method, which involves the crystallization of substances from high-temperature aqueous solutions under high vapor pressures, is another effective technique for nanoparticle synthesis. But the requirement for high-pressure reactors makes it less cost-effective for large-scale production [17]. Zinc oxide nanoparticles, prepared using hydrothermal methods, have demonstrated excellent catalytic properties and have been effectively used for transesterification in biodiesel production [39]. Similarly, zirconium-oxide-based catalysts synthesized hydrothermally have exhibited high acidity and basicity, making them suitable for biodiesel production from low-grade crude palm oil [40].

#### 3.1.3. Precipitation Method

Precipitation techniques are commonly employed for synthesizing nanoparticles, particularly for biodiesel catalysis. For instance, cerium oxide nanoparticles synthesized via the precipitation method have shown excellent performance as a catalyst, achieving a high biodiesel yield and maintaining stability over multiple reaction cycles [41].

#### 3.1.4. Green Synthesis Approaches

Green synthesis methods, which use biological systems such as plant extracts or waste materials, are gaining popularity due to their environmental friendliness. Copper oxide nanoparticles synthesized using plant-based green methods have been applied as additives in biodiesel production, showing significant improvements in engine performance and emission reductions [42].

Plant-derived materials like Garcinia gummi-gutta seed extract have been used to synthesize zinc oxide nanoparticles, which have been evaluated for their catalytic activity in biodiesel production. These green methods are advantageous as they reduce the use of harmful chemicals and promote sustainable production [43]. However, challenges such as batch-to-batch inconsistency, lower reaction rates, and difficulty in achieving a uniform nanoparticle size limit its industrial applicability [17].

### 3.2. Surface Modification and Functionalization

#### 3.2.1. Techniques for Enhancing Compatibility with Biodiesel

One of the primary challenges in incorporating nanoparticles into biodiesel is achieving a uniform and stable dispersion. Surface functionalization using organic molecules, surfactants, or polymers helps improve nanoparticle dispersibility in biodiesel. For instance, the functionalization of barium titanate nanoparticles with organosilanes has been shown to enhance their compatibility with biodiesel blends, leading to improved dispersion and thermal stability [44]. Similarly, functionalizing silica nanoparticles with alkyl chains or sulfonic acid groups creates amphiphilic properties, improving their dispersion and catalytic properties [45,46,47,48].

Green synthesis methods have also emerged as an effective approach for surface modification. Functionalized magnetic nanoparticles synthesized using plant extracts and amino-functionalized coatings have been applied successfully to biodiesel production, enhancing enzymatic recovery and reducing synthesis costs [49]. Additionally, calcium oxide nanoparticles, functionalized with biogenic agents, have demonstrated increased stability and compatibility in biodiesel production [50].

#### 3.2.2. Catalytic Coatings and Doping Methods

Catalytic coatings and doping techniques further enhance the activity and selectivity of nanoparticles in biodiesel applications. Metal-doped nanoparticles, such as cerium–zirconium mixed oxides, exhibit a superior oxygen storage capacity and thermal stability, leading to improved combustion and reduced emissions in biodiesel engines [48]. Another study demonstrated that zinc oxide nanoparticles doped with organic acids not only improved catalytic efficiency but also enhanced the thermal properties of biodiesel [49].

Metal–organic frameworks (MOFs) combined with nanoparticles, such as Ag@MOF-801 composites, represent a novel approach to improving biodiesel synthesis. These composites offer high surface areas and catalytic activity, significantly increasing transesterification efficiency and lowering energy consumption during biodiesel production [50].

The functionalization of nanoparticles with enzymes, such as lipase immobilization on iron oxide nanoparticles, has also proven effective for biodiesel production. This approach enhances the catalytic activity and reusability of enzymes, making the process more cost-effective and sustainable [51].

#### 3.2.3. Emerging Trends and Applications

The integration of nanoparticles with advanced functionalization techniques continues to evolve. Recent studies have explored the use of graphene-based nanocomposites and hybrid nanoparticles for improving biodiesel properties and reducing emissions. For example, graphene-oxide-coated nanoparticles exhibited enhanced thermal stability and improved biodiesel combustion characteristics [52].

The surface modification and functionalization of nanoparticles have a transformative impact on biodiesel production and performance. By improving compatibility, enhancing catalytic properties, and incorporating green synthesis methods, these approaches offer promising pathways for achieving energy sustainability and reducing the reliance on fossil fuels.

### 3.3. Challenges in Nanoparticle Synthesis

#### 3.3.1. Scalability and Cost

The synthesis of nanoparticles at the laboratory scale is well-established; however, transitioning these processes to industrial scales poses significant challenges. Wrasman et al. highlighted that colloidal synthesis, while effective at small scales, requires substantial volumes of solvents and surfactants, significantly inflating production costs. To mitigate this, the study proposed solvent recycling methods, enabling multiple rounds of nanoparticle synthesis without compromising quality [53].

In another approach, Maher et al. utilized naturally occurring diatom silica as a source for biodegradable silicon nanoparticles. This scalable and cost-effective method demonstrated a high surface area and strong luminescent properties, suitable for diverse applications [54]. However, despite these advancements, challenges in process consistency and achieving uniform particle sizes remain.

Emerging techniques such as microfluidic-assisted synthesis offer potential solutions. Agha et al. reviewed the use of microfluidic systems, which provide precise control over reaction conditions, enabling the scalable production of nanoparticles with uniform properties [55]. However, the initial setup costs of such systems can be a barrier for widespread adoption.

#### 3.3.2. Environmental and Safety Concerns

The environmental and health implications of nanoparticle synthesis are gaining attention. Many conventional methods rely on hazardous chemicals and high-energy processes, which contribute to environmental degradation. Thakur and Kumar emphasized the necessity of adopting green synthesis approaches in order to mitigate these risks. Their study advocated for the use of bio-based precursors and plant extracts, which reduce toxicity and energy consumption during synthesis [56].

Safety concerns are not limited to production processes but also extend to the application of nanoparticles. Iravani and Zolfaghari reviewed the eco-toxicity of nanomaterials and suggested adopting a “safe-by-design” approach. This involves designing nanoparticles with minimal environmental and biological impact while maintaining functional efficacy [57]. Palkhiwala and Bakshi revisited traditional Ayurvedic practices for nanoparticle synthesis. They proposed combining ancient techniques with modern green chemistry principles to minimize environmental hazards and improve sustainability [58].

#### 3.3.3. Addressing Challenges Through Innovation

To address scalability and cost, Phakatkar et al. developed a flame spray pyrolysis method for the rapid synthesis of high-entropy oxide nanoparticles. This method offers scalability and precise control over the particle morphology but requires a high initial investment [59]. Rodríguez-Otero et al. explored the use of agricultural waste, such as rice husks, to produce silica nanoparticles. This method not only reduces production costs but also supports waste management, making it an environmentally friendly solution [60].

## 4. Characterization of Nanoparticles for Biodiesel

### 4.1. Physicochemical Properties

#### 4.1.1. Particle Size, Shape, and Distribution

The characterization of nanoparticle properties such as the size, shape, and distribution is crucial for their efficacy in biodiesel applications. Techniques like Scanning Electron Microscopy (SEM) and Transmission Electron Microscopy (TEM) are extensively used to analyze these parameters. For instance, Tariq et al. synthesized cadmium oxide nanoparticles for biodiesel production and utilized SEM and TEM to reveal a size range of 20–30 nm, which significantly enhanced the catalytic efficiency [61].

Similarly, Sahabi et al. employed X-ray diffraction (XRD) and TEM to confirm the formation of ZnO nanoparticles in the size range of 13–47 nm. These nanoparticles demonstrated remarkable catalytic properties during biodiesel transesterification from waste frying oil [62].

In another study, Salim et al. investigated ZnO nanoparticles and emphasized the importance of a uniform size distribution for improved catalytic activity. The study used dynamic light scattering (DLS) in addition to SEM to measure particle size consistency, which showed a high uniformity across the sample [63].

#### 4.1.2. Surface Area and Porosity

Surface area and porosity are critical parameters influencing the reactivity and catalytic performance of nanoparticles in biodiesel production. Magnetic alumina-ferric oxide nanoparticles, with a measured surface area of 187 m^2^/g, exhibited enhanced catalytic activity in converting waste cooking oil into biodiesel, demonstrating the direct link between surface properties and reaction efficiency [64]. Similarly, activated carbon synthesized with an exceptionally high BET surface area of 4320.7 m^2^/g, although primarily studied for CO_2_ adsorption, showed significant potential for catalytic applications in biodiesel production due to its extraordinary surface characteristics [65].

The role of porosity in catalytic performance has also been emphasized in studies on nanoporous Al_2_O_3_, where a specific surface area of 120 m^2^/g contributed to its effectiveness as a biodiesel catalyst [66]. Zinc aluminate nanoparticles, characterized by a high surface area and controlled pore distribution, further demonstrated their potential in optimizing biodiesel conversion efficiency [67].

Beyond metal-based catalysts, cellulose–nanomagnetite composites with a surface area of 250 m^2^/g have shown strong catalytic potential, achieving a conversion efficiency of 89.21%. These findings highlight the significance of tailored surface properties in optimizing reaction kinetics and biodiesel yield [68].

### 4.2. Chemical Composition and Stability

#### 4.2.1. Elemental Composition

Understanding the elemental composition of nanoparticles is crucial for assessing their catalytic and oxidative behaviors in biodiesel applications. Characterization techniques such as XRD and EDS play a vital role in evaluating the nanoparticle purity, structural integrity, and suitability for biodiesel synthesis. For instance, the synthesis of ZnO nanoparticles using a solution-based approach demonstrated their effectiveness in biodiesel applications. An XRD analysis confirmed a particle size range of 13–47 nm, while EDS provided detailed elemental composition data, ensuring the nanoparticles’ purity and compatibility with biodiesel blends [62].

Beyond metal oxide nanoparticles, biochar-based catalysts have also shown promise in biodiesel production. The characterization of sodium-functionalized biochar nanoparticles, derived from residual biomass, revealed the presence of effective catalytic sites, optimizing biodiesel synthesis. XRD and FTIR analyses further validated their structural and functional properties, highlighting their potential as renewable and efficient catalysts [69].

#### 4.2.2. Thermal Stability and Oxidation Resistance

Thermal stability and oxidation resistance are key to maintaining nanoparticle efficiency under high-temperature conditions common in biodiesel combustion. Al_2_O_3_ nanoparticles, when blended with biodiesel, have demonstrated a significant improvement in thermal stability, reducing the rate of oxidative degradation. Thermogravimetric analysis (TGA) studies by Ponnusamy et al. showed that Al_2_O_3_ nanoparticles effectively delayed oxidative weight loss, ensuring prolonged fuel stability—a critical factor for high-performance biodiesel blends [70]. The mechanism behind this improvement lies in the high oxygen affinity of Al_2_O_3_, which traps free radicals and prevents the propagation of oxidation reactions in biodiesel. Similarly, SiO_2_ and Al_2_O_3_ nanoparticles in ionic nanofluids have been found to accelerate nitrate-to-nitrite decomposition below 500 °C, highlighting their role in neutralizing oxidative byproducts and stabilizing fuel molecules [71]. These nanoparticles act as oxidation inhibitors, reducing peroxide formation and preventing gumming and sludge accumulation—common issues in biodiesel storage and long-term use.

#### 4.2.3. Innovations in Characterization Techniques

Recent advancements in nanoparticle characterization include hybrid methods. Alikhani et al. introduced silica-coated magnetic nanoparticles for biodiesel production, employing FTIR and SEM for morphological and compositional analysis. Their approach enhanced the catalytic activity and stability of nanoparticles in biodiesel blends, showcasing the potential for scalable biodiesel applications [33]. Yin et al. (2023) investigated tungsten-trioxide nanoparticles (WO_3_) in polymer composites. A TGA analysis revealed superior thermal stability, while XRD confirmed their structural integrity, making them suitable for high-temperature biodiesel processes [72].

### 4.3. Interaction with Biodiesel Components

#### Adsorption and Dispersion Behavior

The interaction of nanoparticles (NPs) with biodiesel components is critical for improving fuel stability and performance. GO nanoparticles, when combined with dispersants such as CTAB and Tween 80, have demonstrated improved dispersion stability in biodiesel blends. This enhancement led to a 5.067% increase in brake thermal efficiency (BTE) and a 6.293% reduction in brake-specific fuel consumption (BSFC) for mahua biodiesel blends, underscoring the role of nanoparticle dispersibility in optimizing fuel performance [18]. Similarly, chromium oxide (Cr_2_O_3_) nanoparticles incorporated into Mesua ferrea biodiesel blends contributed to increased heating values and improved cetane numbers, emphasizing the importance of nanoparticle stabilization in maintaining biodiesel’s physicochemical properties [73].

The ability of nanoparticles to resist agglomeration and maintain a uniform dispersion significantly influences the oxidative and thermal stability of biodiesel. Studies on Al_2_O_3_ nanoparticles have shown that optimized dispersion techniques, such as ultrasonication, enhance biodiesel stability, leading to improved engine performance and reduced emissions [74]. Furthermore, the addition of TiO_2_ nanoparticles and dimethyl carbonate additives has been linked to notable reductions in CO and HC emissions, demonstrating their effectiveness in enhancing combustion properties in biodiesel blends [75].

A practical application of nanoparticle-infused biodiesel was demonstrated with cerium-coated ZnO nanoparticles in soybean biodiesel blends. These nanocatalysts improved fuel properties, resulting in a 20.66% increase in BTE while significantly reducing harmful emissions such as CO and smoke opacity [76].

## 5. Applications of Nanoparticles in Biodiesel

### 5.1. Catalytic Role in Biodiesel Production

#### 5.1.1. Transesterification Catalysis

Nanoparticle-based catalysts have demonstrated remarkable efficiency and reusability in biodiesel production, making them viable alternatives to conventional catalysts. Molybdenum (VI) complexes anchored on silica-coated magnetic nanoparticles have been successfully employed in transesterification, achieving biodiesel yields exceeding 96% under mild conditions. These Fe_3_O_4_@SiO_2_-APTES-MoO_2_ catalysts not only exhibited high efficiency across various feedstocks, including rapeseed oil, but also retained activity over 11 cycles, reinforcing their cost-effectiveness and long-term applicability [77].

Calcium oxide nanoparticles (CaO NPs), synthesized from calcium carbonate, have further demonstrated significant potential in biodiesel synthesis. A high surface area and basicity enabled these catalysts to achieve an over 95% biodiesel yield through the single-step transesterification of rice bran oil, underscoring their efficiency in biomass conversion [78].

Functionalized ZnO nanoparticles also exhibit strong catalytic activity, particularly in biodiesel production from waste frying oil. These catalysts achieved a biodiesel yield of 97.8%, demonstrating high thermal stability and structural integrity as confirmed through XRD and TGA analysis [63]. Collectively, these findings highlight the growing role of nanoparticle-based catalysts in enhancing biodiesel production through improved efficiency, stability, and reusability.

#### 5.1.2. Esterification Catalysis

Sulfonic-acid-supported magnetic nanoparticles have emerged as a promising solution for enhancing the efficiency of biodiesel production through esterification. These nanoparticles offer advantages such as a high catalytic activity, reusability, and operational stability under varying conditions. In a study by Shaker and Elhamifar (2020), Fe_3_O_4_@OS–SO_3_H nanoparticles were synthesized and demonstrated exceptional catalytic performance. The magnetic core (Fe_3_O_4_) allowed for easy recovery using external magnetic fields, while the sulfonic acid groups provided the necessary acidic sites for catalysis. These catalysts facilitated the esterification of carboxylic acids with alcohols under solvent-free conditions, achieving complete conversion rates at relatively low reaction temperatures. This not only reduced energy consumption but also aligned with sustainable processing goals by minimizing the need for harmful solvents [79].

#### 5.1.3. Green and Sustainable Approaches

Eco-friendly nanoparticles synthesized using plant-mediated methods have demonstrated superior catalytic properties, offering both sustainability and high efficiency in biodiesel production. Magnesium oxide (MgO) nanoparticles synthesized using Terminalia chebula seed extract have been shown to facilitate biodiesel production from used cooking oil while achieving ASTM-compliant fuel properties, emphasizing their practical applicability in renewable fuel development [80]. Similarly, the green synthesis of ZnO nanoparticles using Garcinia gummi-gutta extract presents an innovative approach to reducing environmental impact without compromising catalytic efficiency [43].

Beyond their catalytic potential, nanoparticles also exhibit remarkable reusability, making them viable for long-term applications in biodiesel production. Calcium oxide supported on cobalt ferrite has demonstrated stable performance across multiple cycles, with biodiesel yields reaching up to 95% while retaining catalytic activity after six cycles, reinforcing the economic and environmental benefits of nanoparticle-based catalysts [81]. These findings collectively highlight the role of green-synthesized nanoparticles in promoting sustainable biodiesel production through enhanced catalytic performance and durability.

### 5.2. Enhancing Biodiesel Performance

Research on the integration of nanoparticles with biodiesel has led to significant advancements in both combustion efficiency and emission reduction. Various studies have demonstrated the catalytic role of metal-based nanoparticles in enhancing combustion characteristics and mitigating emissions. For instance, aluminum oxide nanoparticles, when blended with waste fish oil biodiesel, act as combustion catalysts that promote complete combustion, improving brake thermal efficiency by 18% [82]. This catalytic behavior not only enhances energy conversion but also contributes to reduced emissions.

The role of nanoparticles in emission reduction is particularly noteworthy. Aluminum oxide nanoparticles help lower NOx emissions by optimizing the combustion temperature while also decreasing CO and HC emissions due to enhanced oxygen availability in the combustion chamber [82]. Similarly, oxygenated additives such as diethyl ether have demonstrated a 30% reduction in CO and a 20% reduction in HC emissions, attributed to improved oxygenation and cooler flame temperatures [83]. These findings highlight the complementary effects of nanoparticles and oxygenated additives in reducing harmful emissions.

Beyond aluminum-based nanoparticles, carbon nanotubes (CNTs) and copper oxide nanoparticles have also shown promising effects. The addition of CNTs to biodiesel blends has resulted in a 25% reduction in HC emissions and an 18% reduction in NOx emissions, demonstrating their potential in addressing emission challenges across different biodiesel generations [84]. Similarly, biodiesel derived from karanja and safflower oils, when blended with copper oxide nanoparticles, has exhibited lower NOx emissions due to the catalytic influence of the nanoparticles, which alters the flame temperature and promotes complete combustion [85].

The exploration of bio-synthesized nanoparticles further underscores their potential in emission control. Studies have reported that bio-derived nanoparticles contribute to NO_X_ and CO reductions of 15% and 12%, respectively, compared to conventional biodiesel [86]. These collective findings emphasize the growing importance of nanoparticle integration in biodiesel applications, reinforcing their role in enhancing fuel efficiency and reducing environmental impact.

### 5.3. Improving Fuel Properties

#### 5.3.1. Thermal Conductivity Enhancements

Thermal conductivity, a key factor influencing engine performance and heat dissipation, can be significantly improved with the incorporation of nanoparticles. For instance, Anish et al. (2022) evaluated biosynthesized nanoparticles in biodiesel and observed enhanced thermal conductivity. This improvement facilitates a more efficient heat transfer during combustion, thus optimizing fuel utilization. Their study highlighted a 20% enhancement in thermal conductivity compared to the baseline biodiesel, making it suitable for high-performance diesel engines [86]. Elkelawy et al. (2020) investigated the addition of silver thiocyanate nanoparticles to diesel–biodiesel blends and reported a marked increase in thermal conductivity [87]. The study demonstrated how nanoparticles can reduce temperature gradients within the fuel, enabling more uniform combustion.

#### 5.3.2. Viscosity Optimization

Viscosity, a measure of a fluid’s resistance to flow, directly impacts the fuel injection and atomization processes. High viscosity can lead to incomplete combustion, while low viscosity may compromise fuel lubrication. Studies have focused on nanoparticles to balance these dynamics. Ge et al. (2022) used spirulina microalgae biodiesel blended with nanoparticles, achieving a significant reduction in viscosity while maintaining compliance with international fuel standards. This reduced viscosity enhanced fuel atomization and ensured a smoother flow through injection systems [88]. Keihani et al. (2018) produced biodiesel from chicken fat with nano-calcium oxide catalysts. The researchers found that blending biodiesel with diesel at ratios such as B25 and B50 effectively optimized the viscosity, ensuring compatibility with conventional diesel engines [89].

In some cases, metal oxide nanoparticles such as TiO_2_, Al_2_O_3_, and ZnO tend to increase biodiesel viscosity at higher concentrations (above 100 ppm) due to particle aggregation and molecular interactions, which can create flow resistance [90]. Similarly, carbon-based nanomaterials, such as carbon nanotubes (CNTs) and graphene oxide, at concentrations exceeding 80–100 ppm, often lead to an increase in viscosity due to strong surface interactions with biodiesel molecules, making proper dispersion a challenge [91].

However, certain nanoparticles, particularly magnetic and catalytic nanomaterials, have been shown to reduce biodiesel viscosity by altering fuel molecular structures. CeO_2_ and ZnO nanoparticles used at optimized concentrations (50–100 ppm) facilitate the breakdown of high-molecular-weight compounds, reducing glycerides and free fatty acids, which directly lowers biodiesel viscosity [92]. Similarly, magnetic nanoparticles, when used as catalysts in heterogeneous transesterification, produce biodiesel with a lower viscosity, particularly at concentrations below 75 ppm, ensuring compliance with fuel standards [90].

Another critical factor influencing the long-term stability of nanoparticle-enhanced biodiesel is dispersion retention. At high concentrations (>100 ppm), nanoparticles tend to settle over time, leading to phase separation and sedimentation, which negatively impacts fuel consistency. To address this, ultrasonication, surfactants (e.g., cetyltrimethylammonium bromide), and functionalized nanoparticles have been explored as effective strategies for maintaining a uniform dispersion [17]. Studies have shown that nanoparticles stabilized using surfactants remain well-dispersed for up to 30 days, while untreated nanoparticles begin to aggregate within a few days, affecting biodiesel performance [91].

#### 5.3.3. Enhancing Energy Density

Energy density, which determines the amount of energy stored per unit of fuel, is a critical metric for evaluating the suitability of biodiesel for long-haul transportation. Elkelawy et al. (2021) explored 3D silver thiocyanate nanoparticles in biodiesel blends and demonstrated a 15% improvement in energy density [93]. This enhancement was attributed to the nanoparticles’ catalytic activity, which facilitated a more complete combustion of the fuel mixture. Mukhtar et al. (2022) reviewed heterogeneous catalysis for biodiesel production and highlighted the potential of advanced catalysts to increase the calorific value of biodiesel. The improved energy density not only enhances fuel efficiency but also reduces the overall fuel consumption for extended operations [94].

#### 5.3.4. Multifunctional Benefits of Nanoparticles

The multifunctional role of nanoparticles in enhancing biodiesel properties was further demonstrated by Sateesh et al. (2021), who utilized Al_2_O_3_ nanoparticles to simultaneously improve combustion efficiency and thermal conductivity. Their work provided a comprehensive view of how nanoparticle additives can address multiple performance limitations in biodiesel [95].

Despite these advancements, challenges remain in ensuring the economic feasibility and scalability of nanoparticle production and integration. Researchers, such as Ge et al. (2022), emphasize the need for sustainable and cost-effective synthesis methods for nanoparticles. Additionally, long-term engine tests and lifecycle analyses are critical for evaluating the environmental implications of using nanoparticles in biodiesel [88].

Table 1 provides an overview of various nanoparticles used in biodiesel, highlighting their properties and specific effects.

## 6. Current Challenges and Research Gaps

### 6.1. Toxicity and Environmental Impact of Nanoparticles

The toxicity of nanoparticles poses a critical environmental and health challenge. For instance, Abbasi et al. (2023) analyzed the structural parameters of nanoparticles that contribute to their toxicity, emphasizing the role of size, charge, and surface functionality in cytotoxic effects [103]. Furthermore, Ali et al. (2019) reviewed the bioaccumulation of heavy-metal-based nanoparticles, such as ZnO and TiO_2_, highlighting their persistence in ecosystems and their potential to disrupt biological systems [104]. The challenge lies in mitigating these toxic effects without compromising the performance advantages of nanoparticles.

Buchman et al. (2019) proposed strategies for redesigning nanoparticles to reduce their environmental footprint, suggesting modifications in morphology, surface coatings, and functional groups [105].

### 6.2. Long-Term Stability and Compatibility with Engines

The long-term stability and compatibility with existing engine materials remain critical concerns. Nanoparticles often agglomerate over time, losing their functional properties. Chandran (2020) discussed the interactions of biodiesel blended with nanoparticles and its effects on engine materials, indicating wear and compatibility issues under high-temperature conditions [106].

In a recent study, Miriam et al. (2021) conducted a comprehensive compatibility analysis of biodiesel modified with algal oil and nanoparticles in IC engines, finding that specific nanoparticle types improved thermal stability but required precise concentration controls to avoid engine damage [107].

### 6.3. Need for Standardization in Synthesis and Characterization

The lack of standardization in nanoparticle synthesis and characterization leads to inconsistent results and difficulty in comparative analysis. Altammar (2023) highlighted the need for uniform protocols in the green synthesis of nanoparticles to ensure reproducibility and scalability [108]. The absence of standardized characterization techniques also hampers the validation of their efficacy.

Moreover, Graham et al. (2019) discussed the standardization challenges in the context of spirometry but offered insights into the broader application of standardized approaches for nanoparticle technologies [109].

## 7. Future Perspectives

The incorporation of nanoparticles into biodiesel production and performance enhancement has brought significant benefits. However, the focus now shifts toward future advancements to address environmental concerns, compatibility issues, and expanding applications. This section explores the potential directions in nanoparticle research and development for biodiesel, emphasizing eco-friendly synthesis, integration with advanced biofuels, and the exploration of hybrid nanomaterials.

The future of nanoparticles in biodiesel lies in leveraging their unique properties to create a sustainable and efficient energy landscape. Key areas of focus should include the following:

Policy and Standardization: Establishing international guidelines for the safe and consistent application of nanoparticles in biodiesel production.

Cross-disciplinary Collaboration: Integrating insights from materials science, chemistry, and environmental engineering to drive innovation.

Life Cycle Assessment: Conducting comprehensive studies to evaluate the long-term environmental and economic impacts of nanoparticle-enhanced biodiesel.

Advances in these areas could pave the way for transformative changes in the biodiesel industry, offering a cleaner, greener alternative to fossil fuels while addressing global energy demands.

Looking ahead, future research must focus on eco-friendly synthesis methods, leveraging biological materials and green chemistry principles to minimize environmental risks. The integration of nanoparticles with advanced biofuels, such as second-generation biodiesel derived from lignocellulosic biomass, offers an exciting avenue for enhancing the sustainability and efficiency of renewable fuels. Furthermore, the development of hybrid nanomaterials with multifunctional properties could provide innovative solutions to biodiesel’s limitations, such as improved thermal stability and reduced engine wear.

## 8. Conclusions

This comprehensive review has highlighted the transformative potential of nanoparticles in biodiesel applications. Nanoparticles, with their exceptional catalytic properties and ability to enhance fuel characteristics, have demonstrated their role in addressing biodiesel’s challenges, such as the low energy density, poor oxidative stability, and high emissions.

The incorporation of nanoparticles into biodiesel has yielded significant advancements, as evidenced by the improvements in emissions reduction, fuel performance, and environmental sustainability, with notable findings outlined below:

- Nanoparticles are prone to agglomeration over time, leading to a loss of functional properties and reduced performance in real-world applications;
- Eco-friendly synthesis methods, such as plant-based techniques for ZnO nanoparticles, enhance catalytic efficiency and reduce environmental toxicity compared to conventional methods;
- Integrating nanoparticles into second-generation biofuels, like lignocellulosic biodiesel, can increase the energy density by up to 15% and improve the combustion characteristics;
- Hybrid nanomaterials, such as Al_2_O_3_–SiO_2_ composites, increase brake thermal efficiency by 10% and reduce brake-specific fuel consumption by 7%.

Continued research and innovation in this field can revolutionize the energy industry, providing a cleaner, greener alternative to fossil fuels while addressing the pressing challenges of climate change and energy security.

## Figures and Tables

**Table 1:** Characteristics of various nanoparticles in biofuel and their effects.

Nanoparticle	Characteristics	Effects in Biofuel	Ref.
Aluminum Oxide (Al_2_O_3_)	High thermal conductivity, good dispersion, catalytic activity	Improves thermal conductivity and combustion efficiency, reduces emissions	[92]
Silver Thiocyanate	High catalytic efficiency, excellent thermal conductivity	Enhances energy density, improves combustion efficiency, reduces harmful emissions	[87]
Cerium Oxide (CeO_2_)	Oxygen storage capacity, redox properties	Reduces NOx and particulate emissions, improves fuel stability	[96]
Carbon Nanotubes (CNTs)	High surface area, excellent thermal and electrical conductivity	Enhances thermal conductivity, reduces HC emissions, improves energy efficiency	[84]
Titanium Dioxide (TiO_2_)	Photocatalytic properties, high chemical stability	Improves viscosity, enhances combustion characteristics, reduces smoke opacity	[97]
Zinc Oxide (ZnO)	Antimicrobial properties, good thermal conductivity	Reduces NOx and CO emissions, improves oxidative stability of biodiesel	[98]
Iron Oxide (Fe_3_O_4_)	Magnetic properties, high stability under varied temperature	Enhances viscosity control, improves fuel atomization, reduces fuel consumption	[99]
Silica Nanoparticles	High surface area, thermal insulation properties	Reduces friction, improves thermal stability, and enhances the energy density of biofuel	[100]
Magnesium Oxide (MgO)	High heat capacity, excellent thermal stability	Enhances thermal conductivity, reduces unburnt hydrocarbon emissions, and stabilizes fuel during storage	[88]
Nickel Oxide (NiO)	High oxidation resistance, catalytic activity	Improves energy density and reduces engine deposits	[101]
Copper Oxide (CuO)	Excellent thermal conductivity, catalytic properties	Enhances combustion characteristics, reduces CO and HC emissions	[102]

## Data Availability

No new data were created or analyzed in this study. Data sharing is not applicable to this article.
